# First report of *Melittobia australica* Girault in Europe and new record of *M. acasta* (Walker) for Italy

**DOI:** 10.3897/zookeys.181.2752

**Published:** 2012-04-06

**Authors:** Antonino Cusumano, Jorge M. González, Stefano Colazza, S. Bradleigh Vinson

**Affiliations:** 1 Dipartimento DEMETRA, Universitá degli Studi di Palermo, Viale delle scienze, 90128 Palermo, Italy; 2 Texas A & M University, Department of Entomology, College Station, Texas 77843-2475, USA

**Keywords:** Parasitoid wasp, *Sceliphron spirifex*, *Osmia* sp., Sicily, Europe

## Abstract

*Melittobia acasta* and *Melittobia australica* are newly recorded from Sicily, Italy, and the second species is reported in Europe for the first time. A short historical background about *Melittobia* parasitoid wasps, their hosts, and distribution, with emphasis in those two species is presented together with illustrations to facilitate their identification. Brief discussion about the presence and possible distribution of the species in Sicily is also included.

## Introduction

*Melittobia* Westwood is a cosmopolitan genus of gregarious ectoparasitoids that primarily attack prepupae of aculeate Hymenoptera, but are also able to parasitize a wide range of hosts from the orders Coleoptera, Diptera and Lepidoptera (Dahms 1984b; González et al. 2004; Matthews et al. 2009). They are frequently associated with mud-dauber wasps (*Sceliphron* spp. and *Trypoxylon* spp.) but some species are frequently found parasitizing several bee species (i.e. *Anthidium*, *Anthophora*, *Apis*, *Bombus*, *Ceratina*, *Chalicodoma*, *Heriades*, *Megachile*, *Osmia*, *Psythirus*, *Stelis*) (González and Terán 1996; González et al. 2004; Maeta 1978; Matthews et al. 2009).

Twelve species are known in the genus (Matthews and González 2008; Matthews et al. 2009). All *Melittobia* species exhibit extreme intrasexual and intersexual polymorphism, where males are blind, and brachypterous, and have highly modified antennae. Females are either brachypterous, and emerge from the pupae containing a large batch of eggs and are ready to mate, or macropterous, and capable of greater dispersal (Cusumano et al. 2010; González and Matthews 2008; Matthews and González 2008; Matthews et al. 2009; Schmieder 1933). Of these, *Melittobia acasta* (Walker), has been known as the only Eurasian species of *Melittobia* but it occurs widely in other regions of the world (Summarized by González et al. 2004). It has been reported as a dangerous threat to honeybees and/or solitary bees used as crop pollinators (See González and Matthews 2005).

*Melittobia australica* Girault, was described on the basis of three males and ten females that emerged from *Pison spinolae* Shuckard (Hymenoptera: Sphecidae) (Girault 1912). This parasitoid is a nearly cosmopolitan species that has been reported from Australia and New Zealand, several countries of Africa, Asia, North, Central and South America, including a few Caribbean islands/countries (Assem et al. 1982; Dahms 1984a; González and Matthews 2005; González and Terán 1996; González et al. 2008; Maeta 1978).

Here we confirm the presence of two *Melittobia* species (*Melittobia australica* and *Melittobia acasta*) in Italy and provide the first record of *Melittobia australica* from Europe.

## Materials and methods

Collections of trap nests and mud dauber nests were carried out during 2010 in western Sicily. Trap nests were built by pooling together about 10 pieces (Ø=2-3 cm; length=20 cm) of reed (*Arundo donax* L.) that were hung on trees located both in cultivated and uncultivated fields. Nests of mud dauber wasps were collected mainly from external walls of buildings especially in areas close to water sources.

A number of *Melittobia* wasps emerged from trap nests colonized by *Osmia* bees originally placed in the Palermo University campus (38°06’26”N,13°21’07”E). The collected parasitoids were mounted and later identified.

One of several mud dauber nests constructed by *Sceliphron spirifex* L. (Hymenoptera: Sphecidae) collected in the town of Contessa Entellina (province of Palermo) (37°44'23"N, 13°08'27"E), had a cell containing a prepupae of its host parasitized with *Melittobia* wasps. Some were collected and placed on calliphorid pupae (*Calliphora* sp., Diptera: Calliphoridae) to be reared, while the rest were studied and identified. The parasitoids that emerged from these cultures were studied, identified and counted (Table 1).

**Table 1.** *Melittobia australica* Girault emerged from six pupae of *Calliphora* sp., under laboratory conditions (25°C; 75% RH) at Dipartimento DEMETRA, Universitá degli Studi di Palermo, Sicily, Italy

**Host number**	**Males**	**Brachypterous females**	**Macropterous females**	Larvae/Pupae*
1	3	1	60	20
2	2	3	45	28
3	2	1	11	82
4	1	0	5	89
5	4	0	12	68
6	3	1	68	18
Total	15	6	201	305
Mean	2.5	1	33.5	50.83

* These larvae/pupae died during development.

A thorough review of literature was done in order to corroborate that the identified species indeed constituted a new record for the country. We were unable to review other insect collections besides the one at Universitá degli Studi di Palermo, and that of an amateur entomologist in Palermo. Voucher specimens of both parasitoid species and the hosts are deposited at the TAMU insect collection, College Station, Texas, USA and at Palermo University, Palermo, Sicily, Italy.

## Results and discussion

Four males and 63 macropterous females of *Melittobia acasta* were collected (and mounted) from a cell of *Osmia* sp. inside a trap nest.

A total of one male and 376 females of *Melittobia australica* emerged from a *Sceliphron spirifex* prepupa. It is worth noting that the factitious host *Calliphora* pupae appear to be highly nutritious and large allowing the development of brachypterous females (Table 1). From six cultures established using *Calliphora* pupae (four females/fly pupae) as hosts, fifteen males of *Melittobia australica*, as well as six brachypterous females, 201 macropterous females and 305 pupae/larvae emerged (see Table 1).

**Figure 1.**
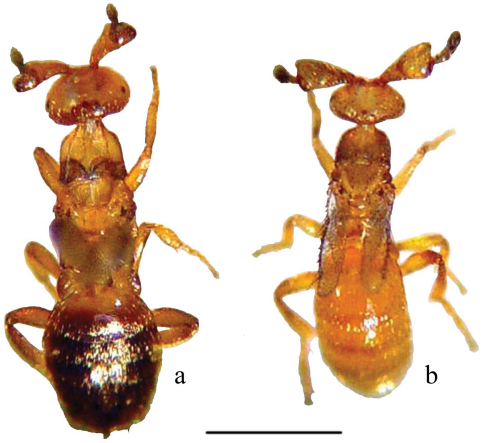
Males of *Melittobia* parasitoid wasps: **a** *Melittobia acasta* **b** *Melittobia australica*. Scale: 0.5 mm

**Figure 2.**
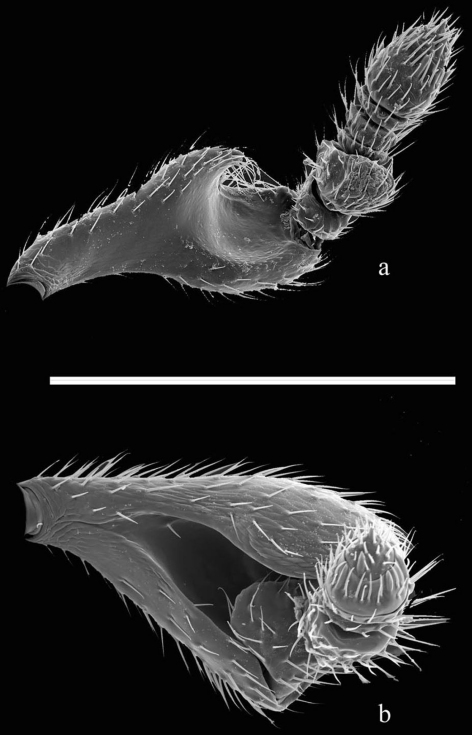
Male antennae of *Melittobia acasta* **a** and *Melittobia australica* **b**. Scale: 0.25 mm.

The easiest way to separate *Melittobia acasta* and *Melittobia australica* is by examining male specimens under a magnifying glass (10×–20×) or a microscope. Males of *Melittobia acasta* are 1.3–1.5 mm long, light brown or amber in color (Figure 1a); antennal scape has a cup-shaped depression, the distal region of the scape is strongly oblique with a broad excavation (Figure 2a) (Dahms 1984a; González and Terán 1996). Males of *Melittobia australica* are 1.1–1.3 mm long, light amber in color and have an antennal scape with a deep ventral longitudinal groove (Figures 1b, 2b) (Dahms 1984a; González and Terán 1996). In order to identify species using females we recommend following Dahms (1984a).

Even though the North American species *Melittobia chalybii* Ashmead was once mentioned in an European country (Denmark) by Holm (1960), it was later clarified that the species was actually *Melittobia acasta* (Holm and Skou 1972; González and Matthews 2005). Otherwise, all reports we had been able to find of the presence of *Melittobia* in European countries, indicate that the species found was always *Melittobia acasta* (González and Matthews 2005; González et al. 2004). Thus it was not surprising that the first *Melittobia* we encountered in Sicily was *Melittobia acasta*. Since the species was encountered in Palermo, a major port-city in the island, we might suspect that the species found its way to Sicily through the many ships that come from many lands to this place. Since *Melittobia acasta* is widely distributed in Europe and Asia, we might even speculate that its invasion of Sicily could have occurred centuries ago.

Even though *Melittobia australica* is a nearly cosmopolitan species, it has never been reported from Europe until now. The finding of this species in the town of Contessa Entellina, located about 80-90 km from the port of Palermo raises an interesting question: how long has it been in Sicily? Since the place where we found *Melittobia australica* in Sicily is in the interior of the island, we might suspect that the species is widely distributed on the island. It is important to note that several species of bees and wasps known from Sicily (Incalterra et al. 2003; Rasmont et al. 2008; Schmid-Egger 2003) are suitable hosts for both *Melittobia* wasps found, helping them to easily establish on the island.
